# Exploiting the Potential of Supported Magnetic Nanomaterials as Fenton-Like Catalysts for Environmental Applications

**DOI:** 10.3390/nano11112902

**Published:** 2021-10-29

**Authors:** Jorge González-Rodríguez, María Gamallo, Julio J. Conde, Zulema Vargas-Osorio, Carlos Vázquez-Vázquez, Yolanda Piñeiro, José Rivas, Gumersindo Feijoo, Maria Teresa Moreira

**Affiliations:** 1 CRETUS Department of Chemical Engineering, Universidade de Santiago de Compostela, 15782 Santiago de Compostela, Spain; maria.gamallo.miron@gmail.com (M.G.); julio.conde@usc.es (J.J.C.); gumersindo.feijoo@usc.es (G.F.); maite.moreira@usc.es (M.T.M.); 2 Laboratory of Magnetism and Nanotechnology, Departments of Physical Chemistry, Faculty of Chemistry, and Applied Physics, Faculty of Physics, Universidade de Santiago de Compostela, 15782 Santiago de Compostela, Spain; zulema.vargas@tnuni.sk (Z.V.-O.); carlos.vazquez.vazquez@usc.es (C.V.-V.); y.pineiro.redondo@usc.es (Y.P.); jose.rivas@usc.es (J.R.); 3 Centre for Functional and Surface Functionalized Glass, Alexander Dubček University of Trenčín, Študentská 2, 91150 Trenčín, Slovakia

**Keywords:** Fenton, nanoparticle, kinetic, SBA-15, estrogen, reuse, magnetic catalyst

## Abstract

In recent years, the application of magnetic nanoparticles as alternative catalysts to conventional Fenton processes has been investigated for the removal of emerging pollutants in wastewater. While this type of catalyst reduces the release of iron hydroxides with the treated effluent, it also presents certain disadvantages, such as slower reaction kinetics associated with the availability of iron and mass transfer limitations. To overcome these drawbacks, the functionalization of the nanocatalyst surface through the addition of coatings such as polyacrylic acid (PAA) and their immobilization on a mesoporous silica matrix (SBA15) can be factors that improve the dispersion and stability of the nanoparticles. Under these premises, the performance of the nanoparticle coating and nanoparticle-mesoporous matrix binomials in the degradation of dyes as examples of recalcitrant compounds were evaluated. Based on the outcomes of dye degradation by the different functionalized nanocatalysts and nanocomposites, the nanoparticles embedded in a mesoporous matrix were applied for the removal of estrogens (E1, E2, EE2), accomplishing high removal percentages (above 90%) after the optimization of the operational variables. With the feasibility of their recovery in mind, the nanostructured materials represented a significant advantage as their magnetic character allows their separation for reuse in different successive sequential batch cycles.

## 1. Introduction

Advanced oxidation processes (AOPs) are based on the formation of reactive oxygen species (ROS) that lead to the non-specific oxidation of pollutants present in wastewater. These non-selective species possess a high oxidation potential with the ability of removing a wide variety of organic molecules, such as pharmaceutically active compounds (PhACs), endocrine disruptors (EDCs) and personal care products (PPCPs), identified as a potential threat to wildlife and humans when released into the environment [1,2]. Although these types of contaminants are usually detected in wastewater effluents in the range of parts per billion, adverse effects on receiving ecosystems have been demonstrated even at low concentrations [3,4]. In this sense, ROS species induce structural changes in their chemical structures, leading to their decomposition and reducing the risks associated with the presence of reaction intermediates in the effluent [5].

AOPs include a wide range of different approaches, such as ozone-based processes [6], semiconductor-based photocatalysis [7], Fenton-type reactions [8], or hybrid processes such as photo-Fenton or the peroxone process [9]. Compared to other options, the Fenton catalysis process has several noteworthy advantages, such as high oxidative capacity, low cost of chemicals and operational simplicity. However, several drawbacks have been mentioned that may limit its application: low efficiency at neutral pH and the generation of large quantities of iron sludge when using homogeneous catalysts, which must be managed before the discharge of the final effluent [8,10]. In order to address these limitations, a heterogeneous catalysis approach based on magnetite nanoparticles is proposed, as their superparamagnetic properties allow their simple recovery by means of a magnetic field. Although the environmental implications in the synthesis of this type of nanomaterials must be considered [11], the possibility of their reuse could limit the potential associated impacts. Based on the premise of a nanocatalyst with iron on its surface and magnetic potential, a separation unit must be implemented to allow its use in subsequent reaction cycles, without significant loss of catalytic activity [12,13,14].

Magnetic nano-catalysts must meet some requirements to achieve a viable practical application, i.e., maintaining constant activity over time and adequate physical, chemical and mechanical stability. However, the nature of magnetic nanoparticles (MNPs) results in interparticle interactions, leading to the formation of large aggregates with a lower surface-to-volume ratio [15]. This drawback can be avoided by modifying the surface of MNPs with stabilizing agents to prevent aggregation of nanocatalysts due to steric or electrostatic forces [16]. Besides colloidal stability of the nanoparticles, the coating process can modify the surface of MNPs to prevent oxidation, to modify adsorption, or to increase the reusability. However, the coating might also hinder the catalytic activity of the MNPs. In this line, a recent review compiled the different advantages and limitations of organic coatings for magnetic nanoparticles [17].

Moreover, the immobilization of nanoparticles on a porous solid is of particular interest to facilitate the access of H_2_O_2_ to the active center of the catalyst as well as to improve its dispersion and separability [18]. Some novel approaches studied MNP immobilization onto different supports, such as cellulose nanofibers [19] or polydopamine matrices [20], resulting in improved degradation of organic pollutants and increased stability and recyclability compared to unsupported MNPs. In this context, the mesoporous silica matrix SBA15 has been used as an immobilization support for nanocatalysts in the field of wastewater treatment [21] as well as in organic synthesis [22], due to its porosity, chemical stability and facile and cost-effective synthesis. Its hexagonally arranged uniform channels with narrow pore size distribution, thick pore walls, high surface area and hydrothermal stability make it suitable for the immobilization of nanostructured catalysts.

In this manuscript, the use of different types of MNPs as efficient heterogeneous Fenton-like catalyst for the oxidation of different types of pollutants is explored, considering the influence of different coatings (PAA, PEI and SiO_2_) and a mesoporous support (SBA15) on the efficiency of the process and the stability of the nanoparticles. A first phase of nanocatalyst selection is proposed based on their dye removal capacity in order to define the optimization strategy for those options with the best performance results for the removal of endocrine disrupting chemicals –estrone (E1), 17β-estradiol (E2) and 17α-ethinylestradiol (EE2).

## 2. Materials and Methods

### 2.1. Chemicals

All chemicals were purchased from commercial suppliers. For the synthesis of the nanocatalysts, tetraethyl orthosilicate (TEOS, 98%), polyethyleneimine (PEI, branched, average Mw 25 kDa), polyacrylic acid (PAA, average Mw 2 kDa), triblock copolymer Pluronic P123 (PEO20-PPO70-PEO20), iron(III) chloride hexahydrate (FeCl_3_·6H_2_O, 97%), hydrochloric acid (HCl, 37%), cyclohexane (C₆H₁_2_, 99.8%), Igepal CO-520 (polyoxyethylene (5) nonylphenylether, branched), isooctane (C₈H₁₈, ≥99%) and isopropanol (C_3_H₈O, ≥99.5%) were obtained from Sigma-Aldrich (St. Louis, MI, USA); hydrogen peroxide (H_2_O_2_, 30% *w*/*w*) from Panreac Química SLU (Barcelona, Spain); iron(II) sulfate heptahydrate (FeSO_4_·7H_2_O, 99%), tetramethylammonium hydroxide (TMAOH, ≈10 *w*/*w*) *ortho*-phosphoric acid (H_3_PO_4_, 85%) and ammonium hydroxide (NH_4_OH, 28%) from Fluka (Buchs, Switzerland), ethanol (C_2_H_5_OH, 99.9%) and acetone (C_3_H₆O, ≥99%) were purchased from Fischer (Waltam, MA, USA) and oleic acid (extra pure) from Merck (Kenilworth, NY, USA). Milli-Q deionized water (Millipore, Burlington, MA, USA) was used to prepare the different solutions and reaction media. Hydrogen peroxide (H_2_O_2_, 30%), Reactive Blue 19 (RB19, 50%), methyl green (MG, 85%), estrone (E1, ≥99%), 17β-estradiol (E2, ≥98%) and 17α-ethinylestradiol (EE2, ≥98%) were purchased from Sigma-Aldrich (St. Louis, MI, USA).

### 2.2. Synthesis of Nanostructured Catalysts

#### 2.2.1. Preparation of Sterically Stabilized Magnetite

Sterically stabilized magnetic Fe_3_O_4_ nanoparticles were prepared by coprecipitation of Fe(II) and Fe(III) salts, following the Massart’s method [23]. The synthesis procedure required 12.15 g of FeCl_3_·6H_2_O (45 mmol) and 8.35 g of FeSO_4_·7H_2_O (30 mmol), with a molar ratio of Fe(III)/Fe(II) ≈ 1.5, mixture to which 100 mL of 0.01 M HCl solution is added in a 250 mL round bottom flask. Once the salts were dissolved, the temperature was increased to 60 °C and 30 mL of 28% *w*/*w* ammonium hydroxide solution was added to the mixture, with immediate formation of black magnetite nanoparticles. The reaction was allowed to continue for 1 h at 60 °C and the resulting precipitate was acidified to pH 4 with HCl (9% *w*/*w*) and then magnetically separated. The nanoparticles were washed four times with deionized water and finally a 10% *w*/*w* TMAOH solution was added at pH 10 to sterically stabilize the magnetite nanoparticles.

#### 2.2.2. Preparation of Poly(Ethyleneimine)-Coated Magnetite and Poly(Acrylic Acid)-Coated Magnetite Nanoparticles

Poly(ethyleneimine)-coated (Fe_3_O_4_@PEI) and poly(acrylic acid)-coated (Fe_3_O_4_@PAA) MNPs were prepared with 2 g of PEI or PAA during the synthesis of the Fe_3_O_4_ MNPs approximately one minute after the addition of ammonium hydroxide. After the reaction was completed, the sample was cooled down to room temperature, and the pH was adjusted to 4 with HCl. Finally, the MNPs were magnetically separated, washed four times with deionized water and re-dispersed in water.

#### 2.2.3. Preparation of Silica-Coated Magnetite Nanoparticles

This procedure starts with the formation of a water-in-oil microemulsion system [24]. For this purpose, bare Fe_3_O_4_ MNPs were exposed to oleic acid (OA) until flocculation occurred. The OA-coated Fe_3_O_4_ MNPs (Fe_3_O_4_@OA) were washed twice with deionized water and dispersed in cyclohexane. Then, polyoxyethylene(5)nonylphenyl ether (Igepal CO-520) and cyclohexane were mechanically stirred for 15 min before adding Fe_3_O_4_@OA MNPs (0.5% *w*/*w* in cyclohexane). Finally, ammonium hydroxide solution and tetraethyl orthosilicate (TEOS) were added consecutively under stirring to form a transparent red reverse microemulsion until the reaction was complete after 16 h. The core-shell nanoparticles were precipitated with isopropanol (IPA) to interrupt the reverse microemulsion and washed four times with IPA and deionized water. Finally, they were subjected to several cycles of centrifugation (9000 rpm, 15 min) and washing with deionized water until no foam was observed. Finally, the core-shell nanoparticles were redispersed in deionized water until further use.

#### 2.2.4. Preparation of the Fe_3_O_4_@PAA/SBA15 Nanocomposite

The synthetic procedure used to obtain the SBA15 matrix based on the Colilla method [25] has been previously reported [26]. According to the synthesis procedure, a triblock copolymer Pluronic P123 (PEO_20_-PPO_70_-PEO_20_) was dissolved at 35 °C in a mixture of deionized water and HCl. TEOS was then added to give a final molar composition of 1.0 M of SiO_2_, 0.017 M of P123 and 3.4 M HCl. The reaction was continued under magnetic stirring for 24 h, followed by a curing step at 100 °C for 24 h; the resulting gel was collected by filtration. Finally, the product was dried and subjected to different washing cycles with organic solvents to remove the remaining block copolymer.

Fe_3_O_4_@PAA/SBA15 nanocomposites were synthesized as previously described by Vargas-Osorio et al. [27] by incorporating 1 g of the SBA15 mesoporous matrix into a flask containing 19 mL of an aqueous solution of 0.01 M HCl, 1.2 g of hexahydrate ferric chloride and 0.8 g of tetrahydrate ferrous sulphate under mechanical stirring. The temperature was increased to 60 °C and 3.5 mL of ammonium hydroxide and 0.195 g of polyacrylic acid (PAA, Mw 2000) were added to the mixture [23,28]. The reaction was allowed to progress for one hour and the resulting precipitate was acidified to pH 4 with HCl (9%) and then magnetically separated. Finally, it was repeatedly washed with distilled water and ethanol, and dried at 60 °C for a period of 12 h. A simplified scheme of the synthetic route for the preparation of Fe_3_O_4_@PAA/SBA15 is presented in the supplementary information (see Figure S1) for the sake of better understanding of the nanocatalyst preparation process.

### 2.3. Characterization Methods

The morphological study of the materials was characterized by scanning electron microscopy (SEM) using a Zeiss FE-SEM ULTRA Plus microscopy (Oberkochen, Germany) and a JEOL JEM-1011 transmission electron microscopy (TEM, Akishima, Tokio, Japan) using an accelerating voltage of 100 kV. Nanoparticle sizes were calculated from the micrographs using ImageJ software [29], with a minimum number of 100 measurements. The analysis of the crystalline phases was carried out by X-ray diffraction (XRD) on powder samples using a Philips PW1710 diffractometer (Cu Kα radiation source, λ = 1.54186 Å) (Eindhoven, NB, The Netherlands). The measurements were collected in the 2θ angle between 10° and 80°, increasing by 0.020° and a time per step of 5 s. The mesoporous matrix structure was analyzed by low-angle XRD in a PANalytical X’Pert Powder Empyrean, in a 2θ range between 0.25° and 6°, and a step size of 0.01° (5 s per step). Fourier transform infrared (FTIR) spectra were recorded in a Thermo Nicolet Nexus spectrometer using the attenuated total reflectance (ATR) method. Thermogravimetry was carried out using a Perkin Elmer TGA 7 thermobalance. The experiments were carried out under N_2_ at a heating rate of 10 °C min^−1^ up to 850 °C with a final isothermal step at 850 °C during 30 min. Pore size distribution and specific surface area were estimated from N_2_ adsorption–desorption isotherms obtained using a Quantachrome Autosorb IQ2 instrument. Magnetization curves as a function of the applied magnetic field up to 10 kOe were obtained at room temperature with a DMS 1660 vibrating sample magnetometer (VSM). The iron content of MNPs and effluents was determined by inductively coupled plasma optical emission spectroscopy (ICP-OES) using a Perkin–Elmer Optima 3300 DV equipped with an autosampler Perkin-Elmer AS91 (Waltham, MA, USA). Samples were directly measured without solid filtration or digestion. The zeta potential of nanoparticles and nanocomposites was measured at pH 3 using a Zetasizer NanoZS (Malvern Panalytical, Almelo, The Netherlands) equipment using the Phase Analysis Light Scattering (PALS) technique.

### 2.4. Selection of Nanoparticles and Nanomaterials as Catalysts in Fenton-Type Reactions for Dye Oxidation

Considering the non-specific reaction mechanisms of Fenton-based processes, dye removal was considered a good approach for the benchmarking of nanoparticles performance prepared with different coatings and nanocatalyst supports. The operating conditions are set after an initial screening using bare Fe_3_O_4_ MNPs for RB19 removal at different conditions of initial concentrations of H_2_O_2_ (100–500 mg L^−1^) and catalyst (100–750 mg L^−1^), with a fixed pH of 3 (see Figures S2 and S3 of the supplementary information). Based on the optimization results, a solution of Reactive Blue 19 (RB19) or Methyl Green (MG) with an initial concentration of 25 mg L^−1^ was degraded using 200 mg L^−1^ of the different nanoparticles and 100 mg L^−1^ of hydrogen peroxide at pH 3.

Batch experiments were performed at room temperature and 150 rpm in an orbital shaker (C24 Incubator shaker, New Brunswick Scientific, Edison, NJ, USA). The nanoparticles and the target compounds were mixed at the beginning of the experiments to achieve adsorption equilibrium, and the reaction was initiated after the addition of 100 mg L^−1^ of H_2_O_2_. At regular time intervals, absorbance measurements were performed to monitor the variation in the characteristic absorbance of the dyes (RB19, λ_max_ = 592 nm and MG, λ_max_ = 633 nm) using a BioTek PowerWave XS2 micro-plate spectrophotometer. The decolorization yield (%) was determined as the color disappearance rate, calculated by the following equation:

Degradation yield (%) = (C_0_ − C_t_)/C_0_ × 100, (1)

where C_0_ represents the initial dye concentration (mg L^−1^), and C_t_ represents the concentration for a given time (t). Additionally, in order to elucidate the influence of the MNPs on the dye removal, adsorption control experiments without H_2_O_2_ and control experiments with H_2_O_2_ dosage but lacking MNPs were conducted.

The reusability of the catalyst was evaluated in a sequential operation of a 10 mL reactor with magnetic separation of nanoparticles. After each Fenton-type cycle, the liquid fraction was withdrawn by separating the catalyst with an external magnetic field. Fresh medium containing RB19 or MG (25 mg L^−1^) was added to the reactor containing the catalyst for a new cycle. Aliquots were taken at the beginning and the end of each cycle to determine the dye concentration and percentage removal. The stability of the catalyst after five consecutive cycles was evaluated by determining the iron content in the liquid fraction by ICP-OES.

### 2.5. Experimental Design for Estrogen Degradation

The experiments of estrogen degradation were conducted in 10 mL reaction medium containing 100–1000 mg L^−1^ of mesoporous supported catalyst (Fe_3_O_4_@PAA/SBA15) and variable concentrations of the estrogens E1, E2 and EE2 (100–500 µg L^−1^). Batch experiments were performed at room temperature and with continuous orbital shaking (150 rpm), pH values from 3 to 5 and different concentrations of H_2_O_2_ in the range of 200 to 500 mg L^−1^. At regular intervals, aliquots (200 μL) were withdrawn to monitor the removal of estrogens. In addition, the degradation yield (%) was determined using Equation (1), and parallel controls were carried out to evaluate the dye adsorption on the catalyst surface, the influence of H_2_O_2_ and pH stability to quantify the individual contributions.

The concentrations of estrogens were quantified by High Performance Liquid Chromatography (HPLC) at a detection wavelength of 278 nm on a Jasco XLC HPLC equipped with a 3110 MD diode array detector and a Gemini^®^ 3 µm C18 110 Å reverse phase column (150 mm × 4.6 mm) from Phenomenex (supplied by Jasco, Ishikawa, Japan) and an HP ChromNav data processor. The gradient elution flow (0.8 mL min^−1^) started with 20% acetonitrile in water followed by an increase to 90%. The detection limits for the estrogens were 36.9 µg L^−1^ for E1, 29.2 µg L^−1^ for E2 and 36.0 µg L^−1^ for EE2. The correlation coefficients of the calibration lines (R^2^) were greater than 0.99.

## 3. Results and Discussion

### 3.1. Catalyst Characterization

The morphology and average particle size of Fe_3_O_4_ MNPs were analyzed by TEM microscopy (Figure S4). Bare Fe_3_O_4_, Fe_3_O_4_@PEI and Fe_3_O_4_@PAA are mainly spherical shaped particles with average diameters ranging from 7.6 to 10.9 nm (Table 1). The average particle size of Fe_3_O_4_@SiO_2_ MNPs increases to diameters of around 20 nm, evidencing the remarkable effect of silica coating on the particle size, as noted by the color difference detected in TEM micrograph analyses.

**Figure 1:**
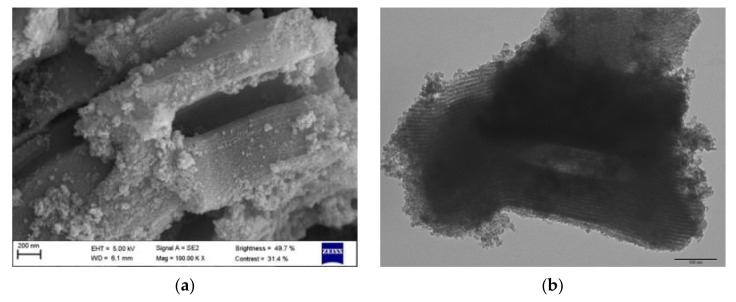
(**a**) SEM and (**b**) TEM micrographs of Fe_3_O_4_@PAA/SBA15. Scale bar on the TEM image corresponds to 100 nm.

The morphology of the Fe_3_O_4_@PAA/SBA15 nanocomposite was also analyzed by SEM and TEM. Considering the synthesis procedure of SBA15, the use of a triblock copolymer (P123) allows to obtain large pore diameters (4–10 nm) and thick walls; thus, producing a stable material. Figure 1 shows the hexagonal structure of the mesoporous silica matrix in which the magnetite nanoparticles have been deposited, forming small aggregates (clear dots) distributed along its surface and predictably within the mesoporous silica channels.

Additional nanoparticle and SBA15 matrix characterization data are presented in Table 1. The size of the mesoporous matrix is in the micrometer range, which is much larger compared to the rest of the nanoparticles considered, which have sizes ranging from 10 to 30 nm. Moreover, the zeta potential of the nanoparticles, essential to predict the stability of the nanoparticles and the interactions between the materials and the target compounds, corresponded to negative values at pH 3 (experimental conditions of the Fenton-based reactions). In the case of the mesoporous matrix, the pore size distribution and the specific surface area were estimated from the type IV adsorption isotherms that are characteristic of mesoporous materials (Figure S5). The boundary hysteresis loop with two-level parallel branches (type H1) confirmed the presence of cylindrical pores in the matrix.

The formation of magnetite by the described synthetic procedures was confirmed by analyzing the crystalline phases present in the MNPs using XRD. Fe_3_O_4_, Fe_3_O_4_@SiO_2_, Fe_3_O_4_@PEI and Fe_3_O_4_@PAA MNPs showed magnetite in crystalline phase as shown in Figure S6, which presents an inverse spinel structure (Fe_3_O_4_, JCPDS PDF-2 card number 19-0629), while Fe_3_O_4_@SiO_2_ showed a broad band between 18° and 29° corresponding to the amorphous silica coating [30]. The structure of the MNPs supported on the mesoporous matrix was evaluated by low-angle XRD (see Figure S7). The alteration of the structural arrangement of SBA15 with the disappearance of (110) and (200) reflections is related to the random distribution of Fe_3_O_4_ MNPs in the nanocomposite, leading to a partial loss of the long-range hexagonal order [31]. PAA coating of the magnetite in the nanocomposite was confirmed by ATR and TGA analysis. Figure S8 show the infrared spectrum of Fe_3_O_4_@PAA/SBA15, showing a characteristic peak between 1700–1720 cm^−1^, assigned to the C=O bond of PAA. The thermogravimetric analysis presented in Figure S9 showed a mass loss of about 12% after water evaporation. However, this mass loss cannot be directly used to quantify the amount of PAA in the nanocomposite, as a small quantity of unwashed surfactant from SBA15 synthesis is being degraded in the same temperature range.

Magnetization studies of Fe_3_O_4_ MNPs and magnetic nanocomposites were performed by measuring the variation of magnetization as a function of the applied magnetic field at 300 K, showing a clear superparamagnetic behavior (negligible coercive forces and remanence) for all samples analyzed (Figure S10). The magnetization properties of the studied MNPs are suitable for magnetic separation from a liquid matrix, being similar to those obtained by other authors in the synthesis of SBA15-based catalysts for Fenton reactions [32].

### 3.2. Preliminary Screening of Nanocatalysts for RB19 and MG Removal

A preliminary screening of different types of magnetite-based nanocatalysts was carried out based on the dye removal performance according to Fenton-type reactions as recommended in different literature reports. Accordingly, a set of experiments is performed on samples containing 25 mg L^−1^ of two model dyes: Reactive Blue 19 (RB19) and Methyl Green (MG), which present different chemical structure and types of chromophores. In particular, RB19 is an anionic anthraquinone dye, while MG is a cationic arylmethane dye. The variation in charge and structure of the molecules informs about the potential of the MNPs to target the removal of compounds with different chemical properties.

The control experiments containing H_2_O_2_ in the absence of a catalyst resulted in low degradation percentages of 13.6% and 1.6% for RB19 and MG, respectively. Following the same trend, the tests evaluating the adsorption of the dyes onto the nanocatalysts showed no significant decrease in dye concentration except for the adsorption of RB19 by PEI-coated magnetite where adsorption is the main contributing factor. Based on the color removal results, the potential of Fenton-type reactions associated with nanomaterials in dye removal is proven. The differences between the removal rates of the degradation and control experiments confirm the suitability of the selected dyes as model compounds.

**Figure 2:**
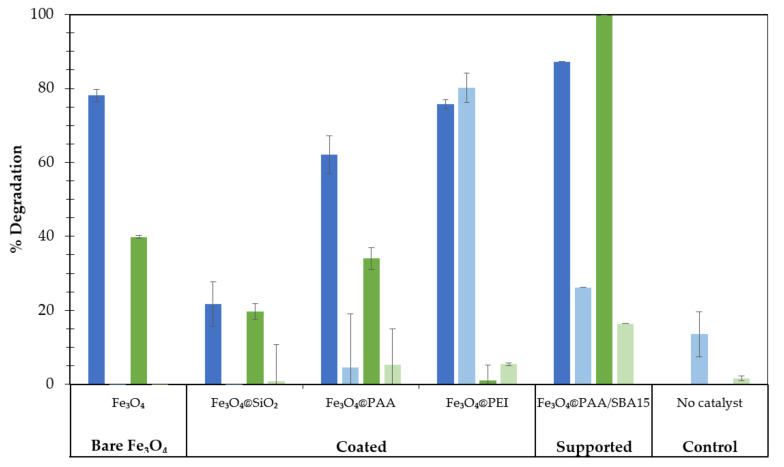
Percentage of dye decolorization (dark bars) and control experiments (light bars) for RB19 after 2 h (blue) and for MG after 4 h of reaction (green), using different Fe_3_O_4_-stabilized MNPs as heterogeneous Fenton catalysts. Operational conditions: [Fe] = 200 mg L^−1^, [H_2_O_2_] = 100 mg L^−1^, pH 3, V = 10 mL.

The effect of the different coatings on Fe_3_O_4_ MNPs (SiO_2_, PAA and PEI) on dye removal was evaluated and compared with bare magnetite, as depicted in Figure 2, resulting in a reduction of the catalytic activity of the bare magnetite. The removal rates obtained using bare nanoparticles were 78% and 40% for RB19 and MG, respectively. When SiO_2_ coating was evaluated, these values decreased to 22% and 20%, with no difference in the adsorption values achieved; thus, this modification significantly decreased the removal values. This decrease is probably caused by the amorphous structure of silica around the nanoparticle limiting molecule access to the catalyst surface, reducing the removal efficiency. Conversely, both PAA and PEI coating increased the adsorption capacity of the catalysts for both dyes, however the removal percentages were lower than those obtained for bare magnetite. The use of polyelectrolytes such as PAA and PEI for nanoparticles covering causes the appearance of electrostatic and steric interactions, that could affect to the dye adsorption and consequently to the removal efficiency. Although the interactions between hydrogen peroxide and covering probably was negligible due to their low molecular weight, these interactions could be magnified for heavier molecules as dyes [33]. Although PEI-coated nanoparticles showed similar removal values in terms of RB19, the decrease in concentration is mainly caused by adsorption and not only by catalytic degradation. From the comparison of the three supports evaluated, the PAA coating resulted in a slight decrease (between 10–15% for both dyes) of the degradation capacity of the bare magnetite, thus resulting in the most suitable strategy for nano-catalyst stabilization.

Differences between the adsorption values of MG and RB19 are probably caused by the differences in zeta potential using different coatings. The effects of coating on MNPs with different types of polymers (such as PAA or PEI) or SiO_2_ alter the surface charge by modifying the interactions between the degradation targets and the catalysts. This phenomenon could explain the high adsorption values of RB19 (negative charge) on PEI-coated magnetite (high positive zeta potential) and the moderate adsorption values for the other type of nanoparticles, with absolute surface charges below 15 mV. Furthermore, negative zeta potential values were reportedly shown to contribute to H_2_O_2_ decomposition, favoring the concentration of H_3_O^+^ near the surface and promoting radical formation [34]. However, the high interaction between PEI-coated magnetite and RB19 is not related to high degradation rates, as a high adsorption value may lead to saturation of the catalyst surface and consequently, to slower kinetics. According to preliminary results, zeta potential values close to zero and negative seem to enhance the removal of the target compounds.

Based on the dye degradation results, the type of nanoparticle selected was Fe_3_O_4_@PAA, which was subsequently immobilized on the mesoporous matrix. As seen in Figure 2, the use of mesoporous support greatly increases the efficiency of the Fenton process, as the best overall removal percentages for RB19 and MG were achieved using PAA-coated magnetite nanoparticles supported in a mesoporous silica matrix (Fe_3_O_4_@PAA/SBA15). Comparing the results with those of bare magnetite, the main difference is observed for MG removal, reaching a significant increase in performance (around 60% removal), while in terms of RB19 removal, an increase of around 10% was observed when using the mesoporous support. Adsorption control experiments showed a significant increase in adsorption values on SBA15-compatible nanoparticles that could be caused by the enhanced surface area of the mesoporous support. The enhancement of catalysts by immobilization on mesoporous silica was also studied by other authors [32,35], also concluding that the use of a mesoporous support improves the catalytic performance by increasing the surface areas and favoring the dispersity of MNPs.

**Figure 3:**
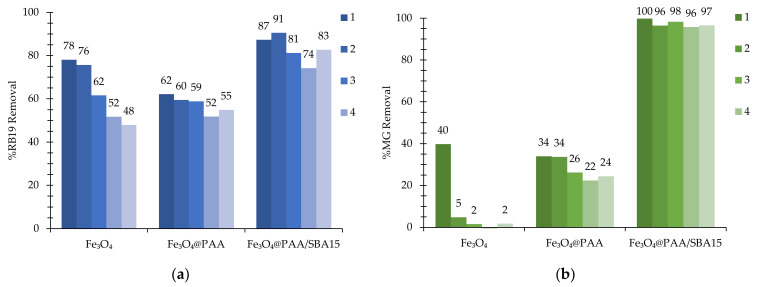
Evaluation of the reusability of Fe_3_O_4_-based MNPs over 5 cycles in the degradation of (**a**) RB19 and (**b**) MG.

One of the requirements to be met by the proposed process is the efficiency of reuse of the MNPs in order to ensure the safe retention of the nanocatalyst and its subsequent use in sequential batch cycles. Degradation efficiency is not the only factor to be considered to ensure the viability of the process, as losses of nanocatalyst after poor separation not only consume an essential resource and affect the economics of the process but may also pose problems of iron presence in the treated effluent and may require a subsequent purification process to avoid the risks associated with the discharge of this type of material. Figure 3 shows the dye removal after five consecutive cycles for bare magnetite, PAA-coated and supported nanoparticles with nano-catalyst recovery performed by external magnetic separation and with no catalyst regeneration between cycles.

Although the addition of PAA as a coating agent shows a lower degradation value in the first cycle compared to bare magnetite, the efficiency of the process in the subsequent cycles is clearly aided by the coating due to the stabilization of the MNPs. The bare magnetite nanoparticles showed a 40% reduction in activity for RB19 removal after 5 cycles, while MG removal under the same conditions decreased to below 5% of the total degradation already by the second cycle. In contrast, the PAA-coated nanocatalyst showed lower decay rates, presenting a 10% and 30% reduction of the removal efficiencies for RB19 and MG, respectively. The addition of a mesoporous silica matrix allowed to increase the removal rates for both RB19 and MG, as well as to reduce the decay values to below 5% when comparing the degradation values for the first and the last cycles.

The recovery of MNPs after the degradation cycles was quantified by analyzing the presence of iron in the treated effluent after the magnetic separation of the effluent after the fifth cycle. Characterization of the effluent after ICP-OES determination provided recovery values of 28.1 ± 3.6% for the bare nanoparticles, these values were increased for the PAA-coating ones to reach a recovery of 86.3 ± 4.1% after five cycles. For the SBA15-supported catalyst, the recovery value of 84.0 ± 2.8% shows a similar performance compared to Fe_3_O_4_@PAA. Although the main factors affecting the recovery of MNPs are their magnetic properties, size and zeta potential, other factors such as agglomeration or matrix composition could influence this factor. Considering iron losses, the most easily separable catalysts were Fe_3_O_4_@PAA and Fe_3_O_4_@PAA/SBA15 nanoparticles. Although the magnetization values of the SBA15-supported nanoparticles are lower than the non-supported ones, their higher size and a similar or lower values of zeta potential could contribute to a better separation.

### 3.3. Heterogeneous Fenton removal for Estrogens Using Fe_3_O_4_@PAA/SBA15

The nanomaterial Fe_3_O_4_@PAA/SBA15 was considered for further characterization studies on estrogen removal due to the excellent results obtained in dye removal experiments in batch operation and in sequential cycles. According to the results obtained under the conditions defined in the previous experiments, the estrogen removal percentages reached levels close to 40% after 6 h of treatment, demonstrating the higher relative stability of this type of compounds against hydroxyl and/or superoxide radicals in the Fenton reaction. Consequently, the reaction conditions for the oxidative degradation of estrogens in the presence of Fe_3_O_4_@PAA/SBA15 were optimized as a function of catalyst and H_2_O_2_ concentration as well as the pH of the reaction medium.

**Figure 4:**
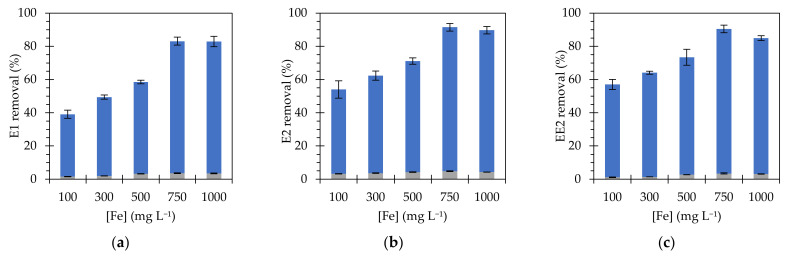
Effect of Fe concentration on the removal of (**a**) E1, (**b**) E2 and (**c**) EE2 (initial concentration of 350 µg L^−1^), using Fe_3_O_4_@PAA/SBA15, after 6 h. Blue bars represent estrogen removal by Fenton reactions; grey bars represent estrogen removal by adsorption.

The effect of Fe_3_O_4_@PAA/SBA15 concentration in the samples was evaluated in the range of 100–1000 mg L^−1^, to degrade E1, E2 and EE2 (C_0_ = 350 µg L^−1^) in the presence of H_2_O_2_ (300 mg L^−1^) at pH 3. The catalytic efficiency was found to increase with catalyst concentration up to 750 mg L^−1^, obtaining degradation rates of 83% (E1), 91% (E2) and 90% (EE2) (Figure 4). Beyond this optimum value, the catalytic efficiency gradually decreased, which can be attributed to the aggregation of the nanocomposite and, consequently, the decrease of the active surface. This behavior was observed by other authors in Fenton-type processes when the catalyst loading reached an upper limit; however, this limit varies depending on different factors such as H_2_O_2_ concentration or catalyst type [36,37]. In addition, high concentration of ferrous ions was observed to be a factor causing the scavenging of hydroxyl radicals [38]. The adsorption control samples showed the same trend, with higher rates at 750 mg L^−1^ of nanocomposite, however the contribution of this parameter to the total degradation is not significant, accounting for 5% of the total removal. The contribution of iron concentration below 750 mg L^−1^ follows a linear trend, showing an average increase of about 10% for each increase of 100 mg L^−1^ in the catalyst concentration.

**Figure 5:**
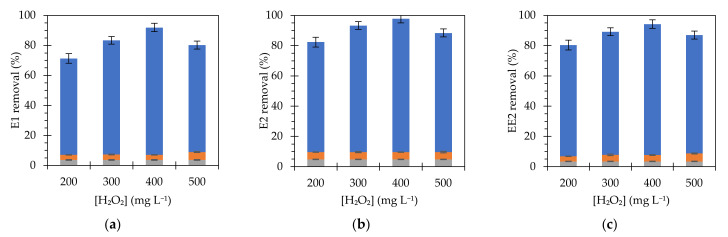
Effect of H_2_O_2_ concentration on the removal of (**a**) E1, (**b**) E2 and (**c**) EE2 (initial concentration of 350 µg L^−1^), using Fe_3_O_4_@PAA/SBA15 with [Fe] at 750 mg L^−1^ after 6 h. Blue bars represent estrogen removal by Fenton reactions; grey bars represent estrogen removal by adsorption; orange bars represent H_2_O_2_ controls.

Optimization of the H_2_O_2_ dosage was studied using the optimal catalyst concentration of 750 mg L^−1^ obtained in the previous step. The results shown in Figure 5 indicate that high concentrations of H_2_O_2_ implied removal percentages lower than expected due to the role of H_2_O_2_ as scavenger in radical production, as previously reported for ferromagnetic nanoparticles [36] and for different types of Fenton catalysts [37,39]. Hydrogen peroxide concentration does not have a large impact on the degradation of estrogens, since variations of 20% were observed between worst and best values, achieved for 200 and 400 mg L^−1^, respectively. The removal percentages have similar values independently of the type of estrogen, reaching slightly higher values for estradiol. In addition, the control experiments provided low degradation yields, showing that the major contribution to the removal is due to the catalytic mechanism. The reactions between the formed radicals and hydrogen caused a scavenging effect and consequently a negative effect on radical production [40,41]. Considering a concentration of 750 mg L^−1^ of catalyst, the best yields were achieved for 400 mg L^−1^ of H_2_O_2_.

**Figure 6:**
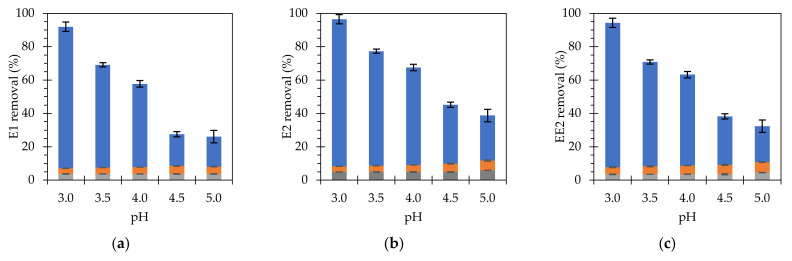
Effect of pH on the removal of (**a**) E1, (**b**) E2 and (**c**) EE2 (initial concentration of 350 µg L^−1^), using [Fe] at 750 mg L^−1^ and H_2_O_2_ at 300 mg L^−1^ after 6 h. Blue bars represent estrogen removal by Fenton reactions; grey bars represent estrogen removal by adsorption; orange bars represent H_2_O_2_ controls.

Finally, the influence of pH was studied to analyze the degradation of target compounds under variable acidic conditions. Although it is well known that Fenton reactions are favored at pH around 3 [10], it is advisable to evaluate the feasibility of increasing the pH of the medium if an environmental application is pursued. Consequently, estrogen removal was investigated at a pH range of 3 to 5 (Figure 6).

A gradual and sustained reduction in removal rates was observed as the pH increased from the optimum value of 3. The total reduction in removal rates was approximately 75% when comparing the initial values with those obtained for pH 5. The control experiments followed a similar trend as for H_2_O_2_ studies. Significant removal rates were not detected at these control tests, concluding that the main removal mechanism is the Fenton catalysis. In concordance to the previous experiments, the elimination percentage observed for E2 was higher than the obtained for E1 and EE2, although the differences are lower compared to the absolute removal ratios. Similar trends were observed by other authors when investigating the influence of pH on the degradation of different types of compounds, using similar degradation mechanisms based on the decomposition of hydrogen peroxide to form hydroxyl radicals. As a general trend, the kinetics decreased significantly when pH values exceeded the upper limit of 4.5 [39,42]. In addition, there were no significant changes in the hydrogen peroxide and adsorption controls under the different experimental conditions.

The optimum result of iron concentration is higher than the values reported by other authors applying homogeneous Fenton [43,44]. The increase of iron concentration is related to the use of heterogeneous catalysts, where the contact between the degradation targets and the iron present in the catalyst is limited. Therefore, the use of higher iron concentrations does not represent a drawback for the application of the process since the magnetic properties of the catalyst allow its reuse and avoid the increase of iron species in the effluent. In addition, another advantage on the use of heterogeneous catalysts is that the increase in pH values does not require the use of stabilizing agents, such as ethylenediaminetetraacetic acid (EDTA) or ethylenediamine-N,N′-disuccinic acid (EDDS), to stabilize the iron in solution. Regarding the ratio of hydrogen peroxide and catalyst concentrations and the results obtained, the optimal ratio is approximately 1:2 of [H_2_O_2_]:[Fe]. When it comes to identifying the contribution of adsorption and H_2_O_2_ addition, the controls showed no significant contribution to the total degradation, with total values below 10%.

### 3.4. Determination of Kinetic Parameters for Estrogen Removal

**Figure 7:**
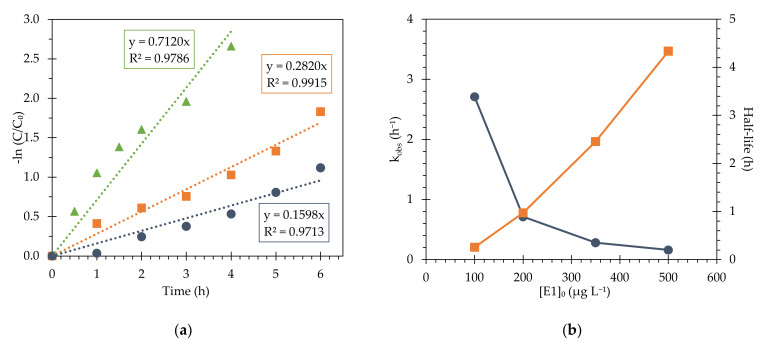
Pseudo-first order fit for E1 removal for (**a**) initial concentrations of 200 (blue circles), 350 (orange squares) and 500 μg L^−1^ (green triangles) and (**b**) kinetic constants (blue circles) and half-life (orange squares) obtained for the degradation experiments conducted at Fe concentrations of 750 mg L^−1^ and H_2_O_2_ at 300 mg L^−1^, at pH 3.

Further analysis of estrogen degradation using Fe_3_O_4_@PAA/SBA15 was performed to determine the kinetic parameters and half-life of each compound. The study of the kinetic parameters will allow identification of the degradation mechanism and comparison with other works and technologies in the field. For this study, 300 mg L^−1^ of hydrogen peroxide and 750 mg L^−1^ of MNP were used under acidic conditions (pH 3.0). The experiments were carried out for 6 h by measuring the concentration at regular intervals, and the data were fitted to a pseudo-first order kinetic model (Figure 7). The R^2^ obtained reached values above 0.97 showing adequate correlation between the experimental data and the model equation. An increasing value of the kinetic constant was observed when the initial concentrations of estrogens were reduced, reaching a E1 half-life of 15 min for an initial concentration of 100 μg L^−1^.

The performance of MNPs versus E2 and EE2 removal considering different initial concentrations are compiled in Table 2 and depicted in Figures S11 and S12 of the Supplementary Information. The results for E2 and EE2 showed a similar trend to that obtained for the degradation of E1, for both the kinetic constants and half-life of the compounds. The highest kinetic value was obtained for the removal of EE2, starting from a concentration of 100 µg L^−1^, being three times lower when the initial target concentration is increased to 200 µg L^−1^. The experimental data fit well by applying a pseudo-first-order model, obtaining correlation coefficients higher than 0.97 for all cases.

As far as the authors are aware, there are not any published results of estrogen degradation by heterogenous Fenton that include the calculation of kinetic constants. Considering other published research works, estrogen removal showed similar kinetic constants compared to other types of micropollutants using coated and supported Fe-based nanoparticles. As an example, Guo et al. [45] reported similar values of a kinetic constant (1.932 h^−1^) studying the degradation of Bisphenol A at pH 3, and using a green-synthesized nanoparticles with a concentration of 300 mg L^−1^. Xin et al. [46] obtained a kinetic constant of 0.2718 h^−1^ using CuFeO_2_/biochar as a catalyst, studying the removal of tetracycline at pH 5 considering an optimum concentration of catalyst of 200 mg L^−1^. The use of carbon nanotubes as support for magnetite also delivered degradation constants in the same order of magnitude for Bisphenol A and methyltestosterone using similar reaction conditions [47,48]. After comparative analysis of kinetic constants, catalyst concentrations and reaction rates, no major differences are observed; however, Fe_3_O_4_@PAA/SBA15 MNPs present, as a unique feature, their excellent recovery and reuse potential. Extending the focus of comparative analysis to other heterogeneous catalytic processes such as photocatalysis using semiconductors (e.g., TiO_2_, ZnO), the reported kinetic values are similar for estrogen removal. Oliveira et al. [49] reported a pseudo-first order rate constant of 0.240 h^−1^ for EE2 removal, which improved to 0.318 h^−1^ when an electro-assisted method was applied. Xu et al. [50] achieved higher reaction rates using magnetically functionalized titanium dioxide for E1 removal under UVC irradiation, obtaining a kinetic constant of 5.1 h^−1^.

## 4. Conclusions

Despite the proven efficacy of heterogeneous Fenton process for micropollutant abatement using nanostructured catalysts, the drawbacks associated with the discharge of iron in the treated effluent imply the need for approaches that allow the retention of the catalyst and its reuse in successive cycles. In this work, the influence of different coatings (PAA, PEI and SiO_2_) was evaluated based on their dye removal capacity. Besides its dye removal ability, PAA-coated magnetite showed an improved stability and catalyst recovery in subsequent cycles compared to bare magnetite nanoparticles. Based on these results, the inclusion of a support is also studied, demonstrating that magnetite nanoparticles coated with polyacrylic acid and immobilized on mesoporous silica matrix support (SBA15) improved the efficiency of heterogeneous Fenton reactions, in terms of removal of dyes and endocrine disrupting compounds, achieving removal efficiencies above 90% using the optimized reaction conditions. The ease of recovery of the mesoporous matrix-supported nanoparticles due to their superparamagnetic properties is noteworthy, increasing their suitability for use in sequential batch cycles and thus reducing the operating costs associated with the loss of the catalyst, showing decay values in removal percentages below 5% and recoverability values up to 84%. The results support the use of magnetite-based magnetic nanocatalysts as a cost-effective alternative to conventional Fenton processes in wastewater treatment for estrogen removal, providing kinetic values in the range of 2.6–3.2 h^−1^ considering the best conditions, similar to other heterogeneous advanced oxidation processes.

## Figures and Tables

**Table 1:** Properties of the different nano-catalysts used in this work.

Catalyst	Size (nm)	S_BET_^1^ (m^2^ g^−1^)	PS_DFT_^2^ (nm)	ZP ^3^ (mV)
Fe_3_O_4_	8.2 ± 3.8	-	-	−11.54
Fe_3_O_4_@PEI	10.9 ± 3.0	-	-	29.94
Fe_3_O_4_@PAA	7.6 ± 2.7	-	-	−4.53
Fe_3_O_4_@SiO_2_	20.2 ± 4.2	-	-	−14.28
Fe_3_O_4_@PAA/SBA15	>1000	276.9	7.59	−1.47

^1^ S_BET_: BET specific surface area, ^2^ PS_DFT_: pore size, ^3^ ZP: zeta potential.

**Table 2:** Kinetic constants and half-life for Fe_3_O_4_@PAA/SBA15 degradation experiments performed in this study compared with other Fe-based nanocatalysts reported in the literature.

Ref.	Pollutant	MNP	Concentration (µg L^−1^)	k_obs_ (h^−1^)	t_1/2_ (h)	R^2^
This work	E1	Fe_3_O_4_@PAA/SBA15	500	0.160 ± 0.011	4.34 ± 0.30	0.9713
			350	0.282 ± 0.011	2.46 ± 0.09	0.9915
			200	0.712 ± 0.043	0.97 ± 0.06	0.9791
			100	2.708 ± 0.241	0.26 ± 0.02	0.9769
This work	E2	Fe_3_O_4_@PAA/SBA15	500	0.228 ± 0.016	3.03 ± 0.21	0.9729
			350	0.366 ± 0.012	1.89 ± 0.06	0.9931
			200	0.898 ± 0.060	0.77 ± 0.05	0.9741
			100	2.613 ± 0.037	0.27 ± 0.00	0.9717
This work	EE2	Fe_3_O_4_@PAA/SBA15	500	0.214 ± 0.011	3.24 ± 0.17	0.9840
			350	0.361 ± 0.015	1.92 ± 0.08	0.9895
			200	0.909 ± 0.054	0.76 ± 0.04	0.9796
			100	3.211 ± 0.271	0.22 ± 0.02	0.9791
[45]	BPA	GS-Fe	25	1.338	-	0.9994
			50	1.050	-	0.9972
			75	0.468	-	0.9985
[46]	TC	CuFeO_2_/biochar	20	0.272	-	-
[47]	BPA	Fe_3_O_4_@MWCNT	70	0.330	-	-
[48]	MT	Fe_3_O_4_@MWCNT	212	0.396	-	0.9420

BPA: Bisphenol-A, GS: grape seed extract (green phenol capping), TC: tetracycline, MWCNT: multi-walled carbon nanotubes, MT: 17α-methyltestosterone.

## Supplementary Materials

The following are available online at https://www.mdpi.com/article/10.3390/nano11112902/s1, Figure S1: Scheme of the synthetic route for the preparation of Fe_3_O_4_@PAA/SBA15, Figure S2: (a) Optimization of H_2_O_2_ concentration using 200 mg L^−1^ of bare Fe_3_O_4_ nanoparticles for the degradation of RB19 with an initial concentration of 30 mg L^−1^ and (b) kinetic fitting. The experimental data correspond to 100 (blue triangles), 200 (orange circles) and 500 (grey squares) mg L^−1^ of H_2_O_2_, Figure S3: (a) Optimization of catalyst concentration (bare Fe_3_O_4_) using 100 mg L^−1^ of H_2_O_2_ for the degradation of RB19 with an initial concentration of 30 mg L^−1^ and (b) kinetic fitting. The experimental data correspond to 100 (blue triangles), 200 (orange circles), 500 (grey squares) and 750 (green diamonds) mg L^−1^ of catalyst, Figure S4: (a) TEM images of Fe_3_O_4_, (b) Fe_3_O_4_@PEI, (c) Fe_3_O_4_@PAA and (d) Fe_3_O_4_@SiO_2_. Scale bars correspond to 50 nm, Figure S5: Adsorption isotherm and pore-size distribution of Fe_3_O_4_@PAA/SBA15 nanocomposite, Figure S6: X-ray diffraction (XRD) characterization of magnetite-based nanoparticles, Figure S7: (a) X-ray diffraction (XRD) characterization and (b) low angle patterns of supported Fe_3_O_4_@PAA/SBA15 nanocomposite, Figure S8: Attenuated Total Reflectance (ATR) spectrum of Fe_3_O_4_@PAA/SBA15 nanocomposite, Figure S9: Thermogravimetric analysis (TGA) of Fe_3_O_4_@PAA/SBA15 nanocomposite, Figure S10: Vibrating sample magnetometer (VSM) curves of bare and coated Fe_3_O_4_ MNPs and Fe_3_O_4_@PAA/SBA15 nanocomposite. The magnetization for coated silica and mesoporous silica nanocomposites was increased by a factor of 10 to improve the visualization of results. Insert figure shows a detail at low applied magnetic fields, confirming the superparamagnetic behavior at room temperature, Figure S11: Pseudo-first order fit for E2 removal for initial concentrations of 200 (blue circles), 350 (orange squares) and 500 μg L^−1^ (green triangles) obtained for the degradation experiments conducted at Fe concentrations of 750 mg L^−1^ and H_2_O_2_ at 300 mg L^−1^, at pH 3, Figure S12: Pseudo-first order fit for EE2 removal for initial concentrations of 200 (blue circles), 350 (orange squares) and 500 μg L^−1^ (green triangles) obtained for the degradation experiments conducted at Fe concentrations of 750 mg L^−1^ and H_2_O_2_ at 300 mg L^−1^, at pH 3.
