# Supplementary material for: Exploiting the Potential of Supported Magnetic Nanomaterials as Fenton-Like Catalysts for Environmental Applications
Source: Nanomaterials (Basel). 2021 Oct 29;11(11):2902. doi: 10.3390/nano11112902 (PMC8617662; doi:10.3390/nano11112902)
Supplement: Supplementary file 1 [file nanomaterials-11-02902-s001.zip › nanomaterials-1432979-supplementary.pdf]

Supplementary material

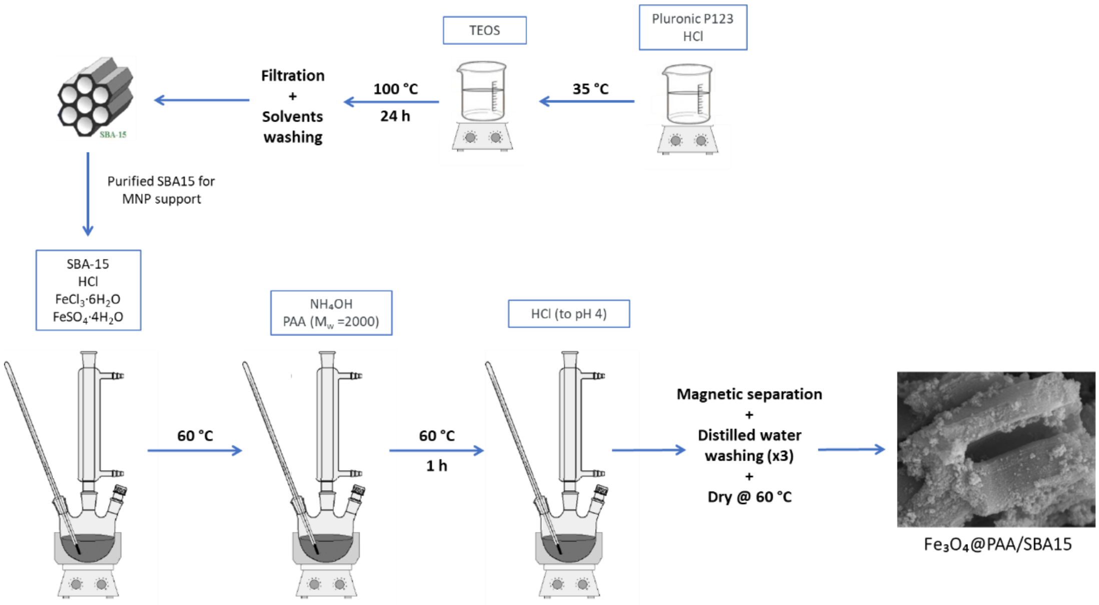

Figure S1. Scheme of the synthetic route for the preparation of Fe_3O_4@PAA/SBA15

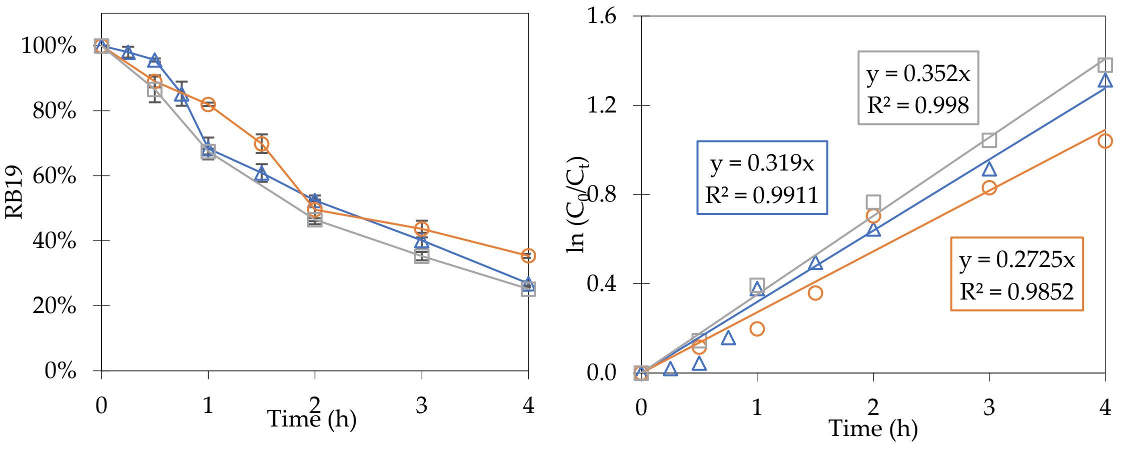

(**a**) (**b**)

Figure S2. (**a**) Optimization of H_2O_2 concentration using 200 mg L^{-1} of bare Fe_3O_4 nanoparticles for the degradation of RB19 with an initial concentration of 30 mg L^{-1} and (**b**) kinetic fitting. The experimental data correspond to 100 (blue triangles), 200 (orange circles) and 500 (grey squares) mg L^{-1} of H_2O_2.

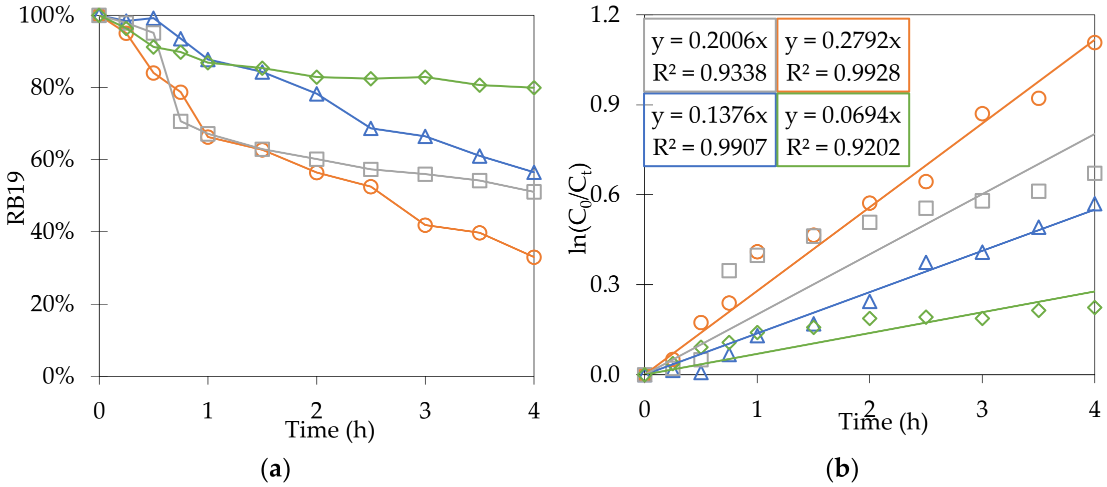

Figure S3. **(a)** Optimization of catalyst concentration (bare Fe_3O_4) using 100 mg L^{-1} of H_2O_2 for the degradation of RB19 with an initial concentration of 30 mg L^{-1} and (**b**) kinetic fitting. The experimental data correspond to 100 (blue triangles), 200 (orange circles), 500 (grey squares) and 750 (green diamonds) mg L^{-1} of catalyst.

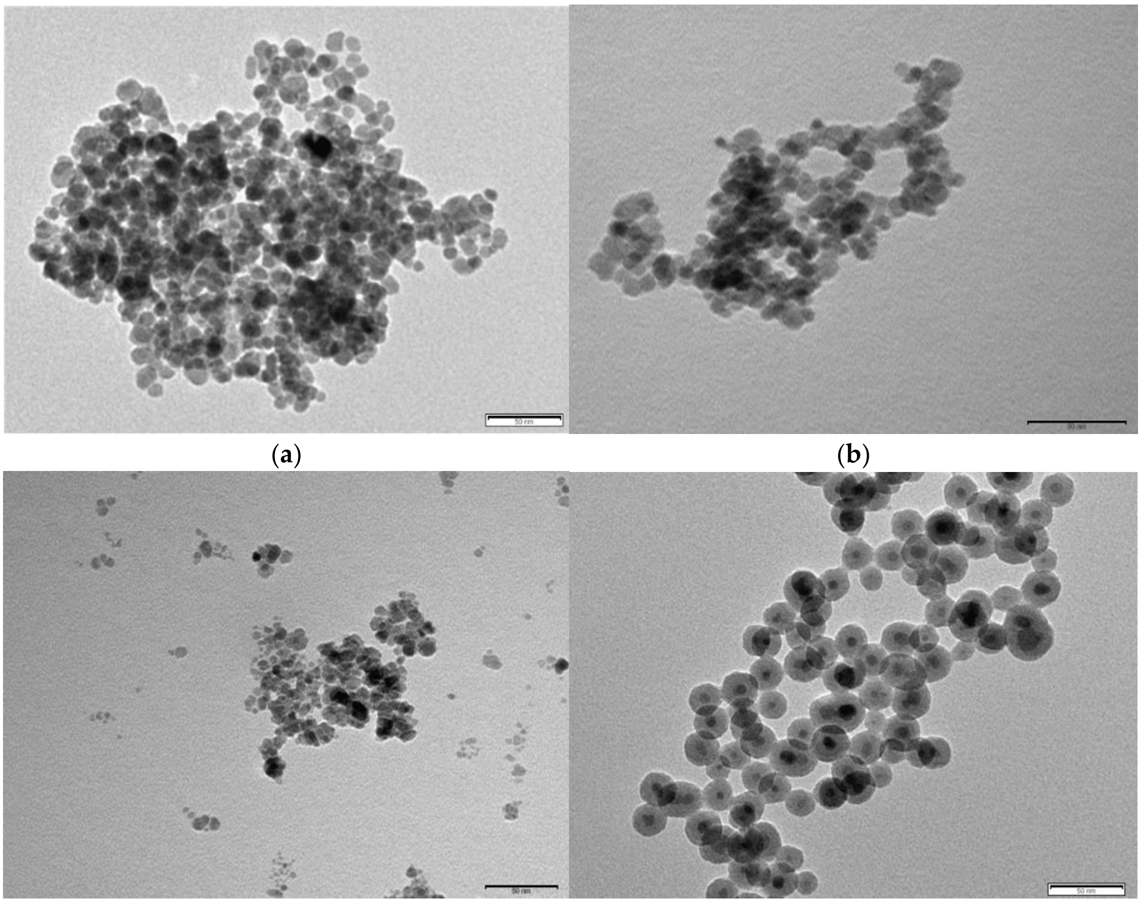

(**a**) (**b**) (**c**) (**d**)

Figure S4. TEM images of (**a**) Fe_3O_4, (**b**) Fe_3O_4@PEI, (**c**) Fe_3O_4@PAA and (**d**) Fe_3O_4@SiO_2. Scale bars correspond to 50 nm.

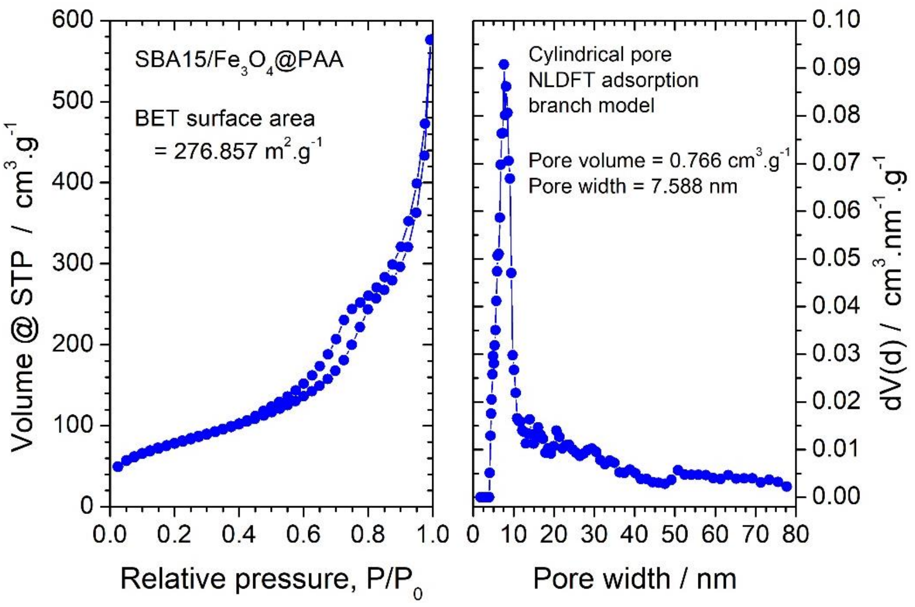

Figure S5. Adsorption isotherm and pore-size distribution of Fe_3O_4@PAA/SBA15 nanocomposite.

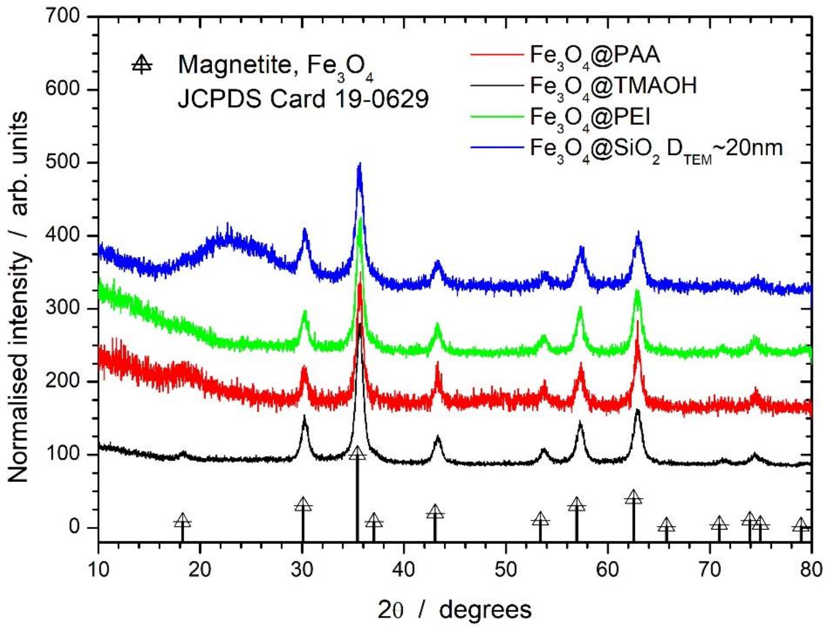

Figure S6. X-ray diffraction (XRD) characterization of magnetite-based nanoparticles.

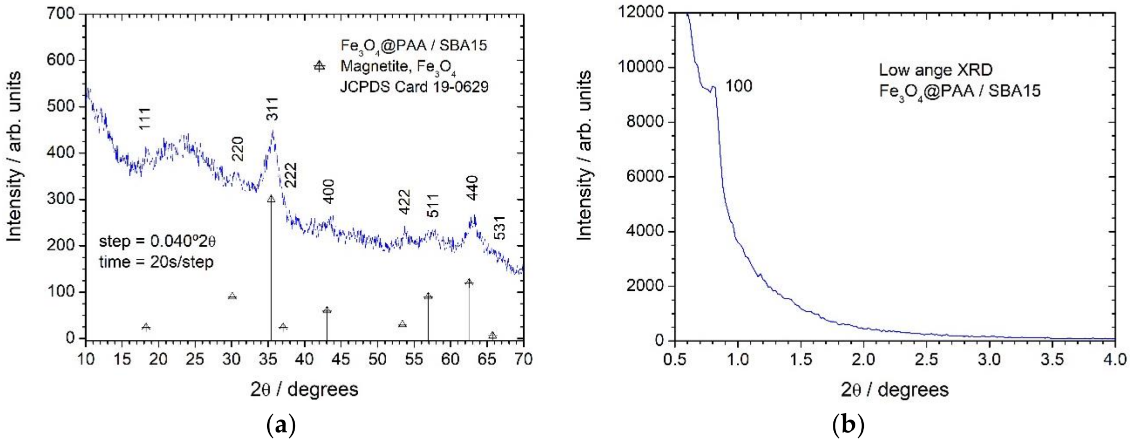

Figure S7. (**a**) X-ray diffraction (XRD) characterization and (**b**) low angle patterns of supported Fe_3O_4@PAA/SBA15 nanocomposite.

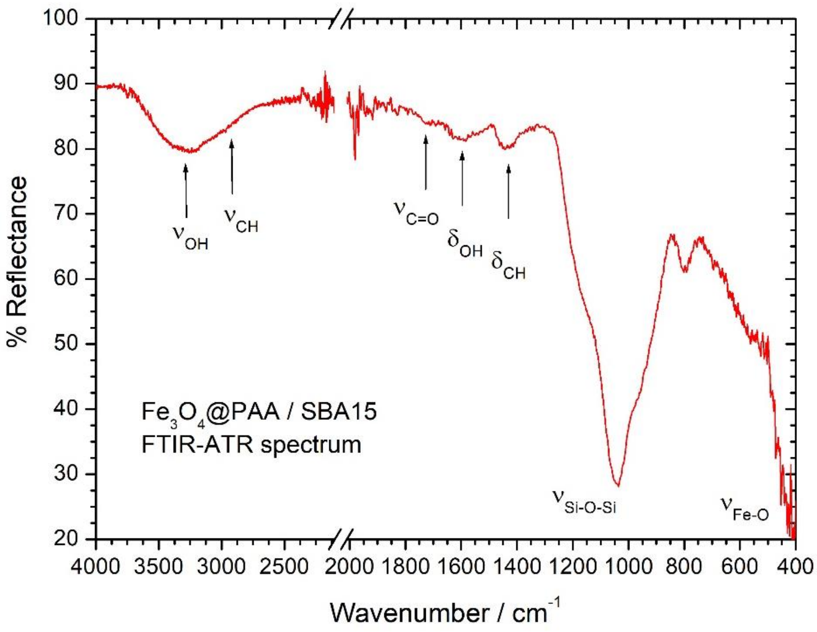

Figure S8. Attenuated Total Reflectance (ATR) spectrum of Fe_3O_4@PAA/SBA15 nanocomposite.

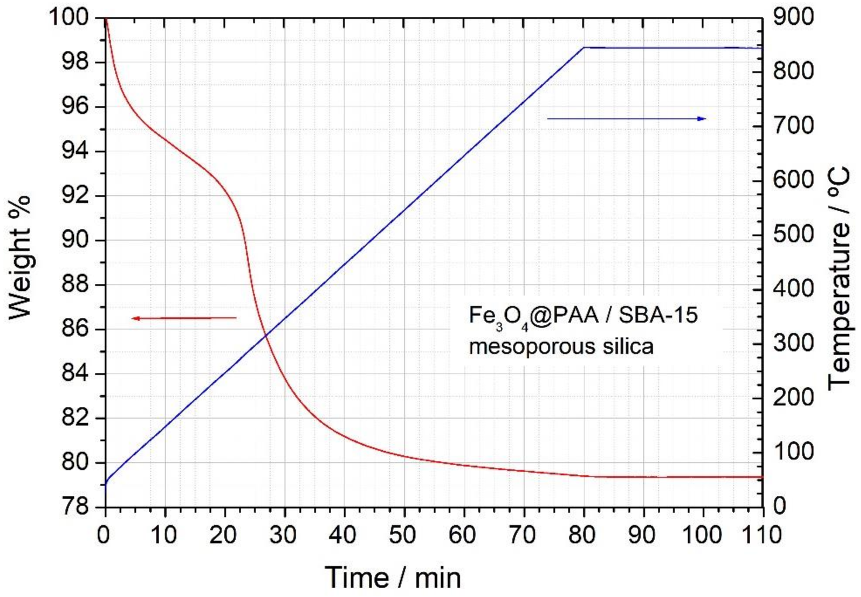

Figure S9. Thermogravimetric analysis (TGA) of Fe_3O_4@PAA/SBA15 nanocomposite.

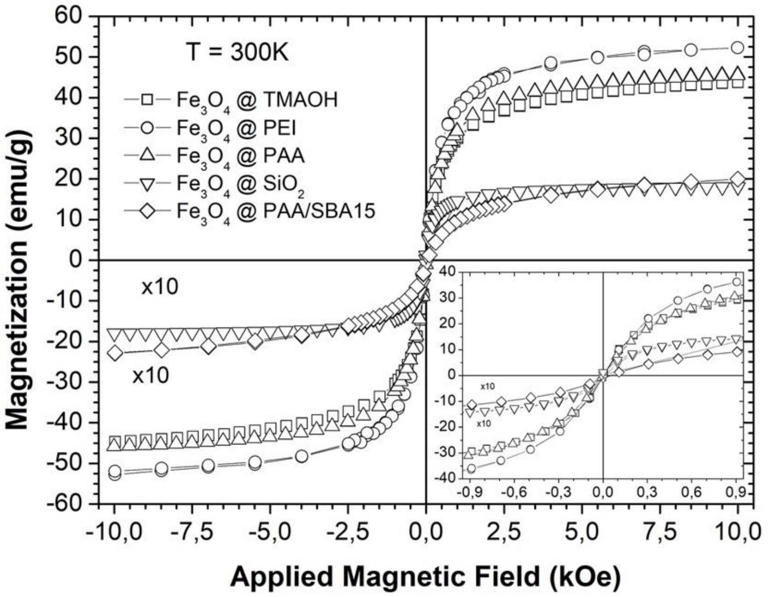

Figure S10. Vibrating sample magnetometer (VSM) curves of bare and coated Fe_3O_4 MNPs and Fe_3O_4@PAA/SBA15 nanocomposite. The magnetization for coated silica and mesoporous silica nanocomposites was increased by a factor of 10 to improve the visualization of results. Insert figure shows a detail at low applied magnetic fields, confirming the superparamagnetic behavior at room temperature.

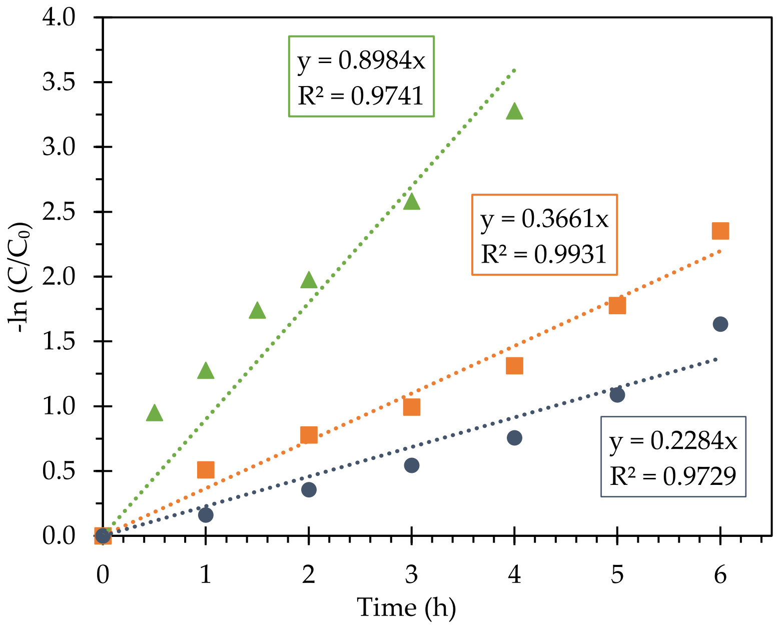

Figure S11. Pseudo-first order fit for E2 removal for initial concentrations of 200 (blue circles), 350 (orange squares) and 500 µg L^{-1} (green triangles) obtained for the degradation experiments conducted at Fe concentrations of 750 mg L^{-1} and H_2O_2 at 300 mg L^{-1}, at pH 3.

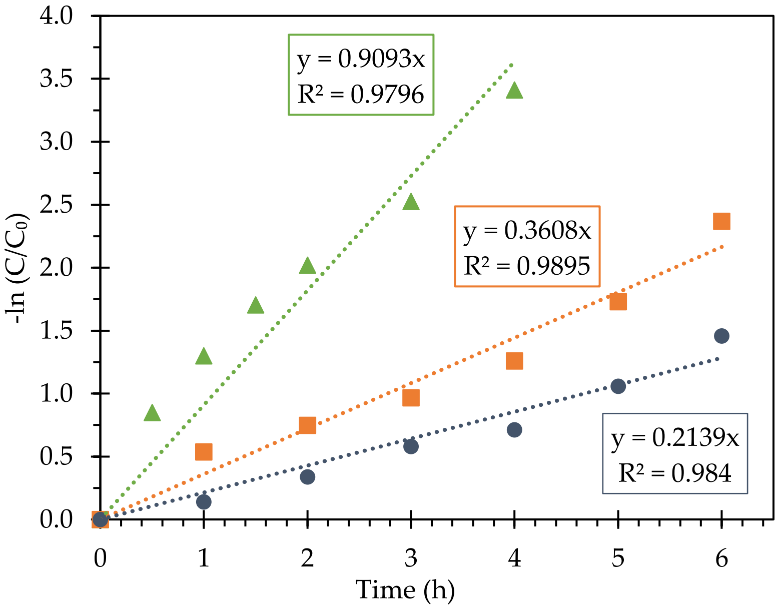

Figure S12. Pseudo-first order fit for EE2 removal for initial concentrations of 200 (blue circles), 350 (orange squares) and 500 µg L^{-1} (green triangles) obtained for the degradation experiments conducted at Fe concentrations of 750 mg L^{-1} and H_2O_2 at 300 mg L^{-1}, at pH 3.
